# A novel *Babesia* sp. associated with clinical signs of babesiosis in domestic cats in South Africa

**DOI:** 10.1186/s13071-019-3395-x

**Published:** 2019-03-26

**Authors:** Anna-Mari Bosman, Barend L. Penzhorn, Kelly A. Brayton, Tanya Schoeman, Marinda C. Oosthuizen

**Affiliations:** 1 Vectors and Vector-borne Diseases Research Programme, Department of Veterinary Tropical Diseases, Faculty of Veterinary Science, University of Pretoria, Private Bag X04, Onderstepoort, 0110 South Africa; 2 Centre for Wildlife Studies, Faculty of Veterinary Science, University of Pretoria, Private Bag X04, Onderstepoort, 0110 South Africa; 3 National Zoological Garden, South African National Biodiversity Institute, PO Box 754, Pretoria, 0001 South Africa; 4 Department of Veterinary Microbiology and Pathology, Washington State University, Pullman, Washington USA; 5 Department of Companion Animal Clinical Studies, Faculty of Veterinary Science, University of Pretoria, Private Bag X04, Onderstepoort, 0110 South Africa; 6 Cape Animal Medical Centre, 78 Rosmead Avenue, Kenilworth, Cape Town, South Africa

**Keywords:** *18S* rRNA gene, *Babesia leo*, *Babesia* sp. cat Western Cape, Domestic cat, Felidae, Phylogeny, South Africa

## Abstract

**Background:**

Feline babesiosis, sporadically reported from various countries, is of major clinical significance in South Africa, particularly in certain coastal areas. *Babesia felis*, *B. leo*, *B. lengau* and *B. microti* have been reported from domestic cats in South Africa. Blood specimens from domestic cats (*n* = 18) showing clinical signs consistent with feline babesiosis and confirmed to harbour *Babesia* spp. piroplasms by microscopy of blood smears and/or reverse line blot (RLB) hybridization were further investigated. Twelve of the RLB-positive specimens had reacted with the *Babesia* genus-specific probe only, which would suggest the presence of a novel or previously undescribed *Babesia* species. The aim of this study was to characterise these organisms using *18S* rRNA gene sequence analysis.

**Results:**

The parasite *18S* rRNA gene was cloned and sequenced from genomic DNA from blood samples. Assembled sequences were used to construct similarity matrices and phylogenetic relationships with known *Babesia* spp. Fifty-five *18S* rRNA gene sequences were obtained. Sequences from 6 cats were most closely related to published *B. felis* sequences (99–100% sequence identity), while sequences from 5 cats were most closely related to *B. leo* sequences (99–100% sequence identity). One of these was the first record of *B. leo* in Mozambique. One sequence had 100% sequence identity with the published *B. microti* Otsu strain. The most significant finding was that sequences from 7 cats constituted a novel *Babesia* group with 96% identity to *Babesia* spp. previously recorded from a maned wolf (*Chrysocyon brachyurus*), a raccoon (*Procyon lotor*) from the USA and feral raccoons from Japan, as well as from ticks collected from dogs in Japan.

**Conclusions:**

*Babesia leo* was unambiguously linked to babesiosis in cats. Our results indicate the presence of a novel potentially pathogenic *Babesia* sp. in felids in South Africa, which is not closely related to *B. felis*, *B. lengau* and *B. leo*, the species known to be pathogenic to cats in South Africa. Due to the lack of an appropriate type-specimen, we refrain from describing a new species but refer to the novel organism as *Babesia* sp. cat Western Cape.

## Background

Domestication of cats occurred in the Near East, probably by natural selection, the ancestor being the local feline subspecies, *Felis silvestris lybica* [1]. From here, domestic cats (*Felis silvestris catus*) have spread world-wide with a current total population of kept or feral cats estimated at nearly one billion [2]. With the exception of Australia, all inhabited continents also harbour indigenous felid species from which pathogens could conceivably be transferred to domestic cats. Feline babesiosis may be a case in point. Although cases of cats showing clinical signs of babesiosis have been reported sporadically from various countries, feline babesiosis seems to be an important disease of domestic cats only in South Africa, especially along the eastern and southern seaboard and with a few foci on the eastern escarpment [3, 4].

*Babesia felis* was described from a *c*.3-month-old wild-caught Sudanese wild cat (*Felis ocreata*, presumably a synonym of a *F. silvestris* subspecies) that was observed for 12 months but showed no overt clinical signs of disease [5]. Parasitaemia, initially 0.5%, soon peaked at 8% (possibly due to stress while the host was adapting to captivity), but gradually decreased over a 3-month period and subsequently fluctuated around 0.4%. Blood from this cat was inoculated into 22 domestic cats. None of these cats showed any overt clinical signs of disease, but all developed a parasitaemia not exceeding 1% initially and then decreasing to a fluctuating low level which persisted indefinitely [5]. Following the classification suggested by Wenyon [6], Davis [5] assigned the novel parasite to the genus *Babesia*; he did not designate and deposit a type-specimen, however, which led to subsequent confusion.

During the 1930s domestic cats exhibiting clinical signs similar to those of canine babesiosis, i.e. anaemia, icterus and lethargy, were occasionally presented to veterinarians in South Africa, especially in the Western Cape Province [7, 8]. *Felis caffra*, presumably the local subspecies of *F. silvestris*, was suspected as being a reservoir host [8]. In the index case report of feline babesiosis [7], the piroplasms seen on blood smears met the description of *B. felis* piroplasms by Davis [5]. Due to its pathogenicity in domestic cats, in contrast to *B. felis* (*sensu stricto*), Jackson et al. [7] proposed the name *Nuttalia felis* var. *domestica* for the South African organism. Choosing *Nuttalia* rather than *Babesia* as genus name, they followed Carpano et al. [9] in preferring the classification by Du Toit [10] rather than that of Wenyon [6].

Regrettably, Jackson’s [7] conclusion that the South African organism represented a distinct taxon to *B. felis* (*s.s*.), being at least a local variety of the latter, was overlooked in subsequent reports on clinical manifestation and treatment of feline babesiosis: the causative organism was merely referred to as *B. felis* [11–13]. This was also the name used when details of molecular characterisation of the *Babesia* sp. causing disease in cats were deposited in the GenBank database [14]. The matter will only be resolved if Davis’s [5] original specimens are traced, which seems unlikely. Molecular characterisation has since revealed the presence of *B. felis* (*sensu lato*) in cheetahs (*Acinonyx jubatus*), lions (*Panthera leo*) and servals (*Leptailurus serval*) in South Africa, Namibia and Zambia [15, 16].

**Fig. 1:**
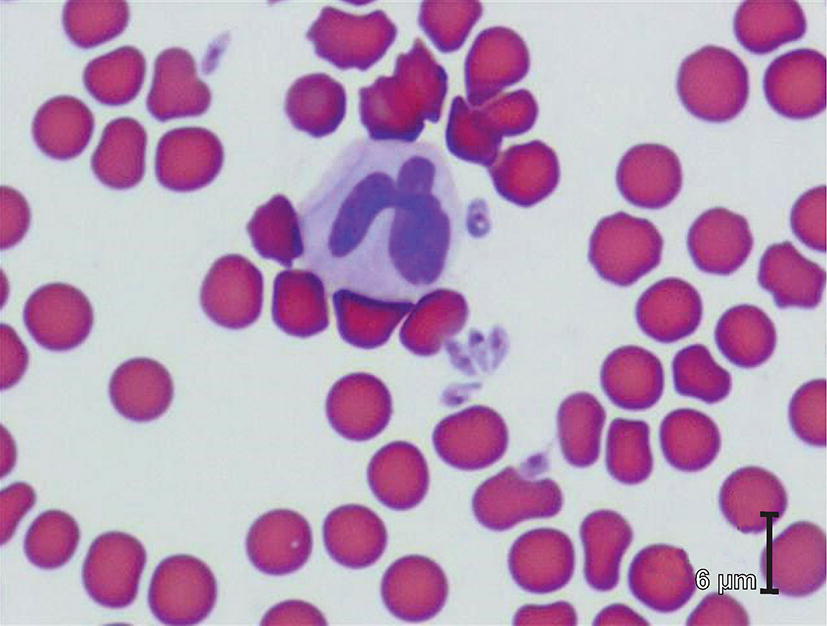
Blood smear from a cat with clinical signs of babesiosis, showing large, extracellular piroplasms (Courtesy: Dr James Hill, Vetdiagnostix, Pietermaritzburg)

**Fig. 2:**
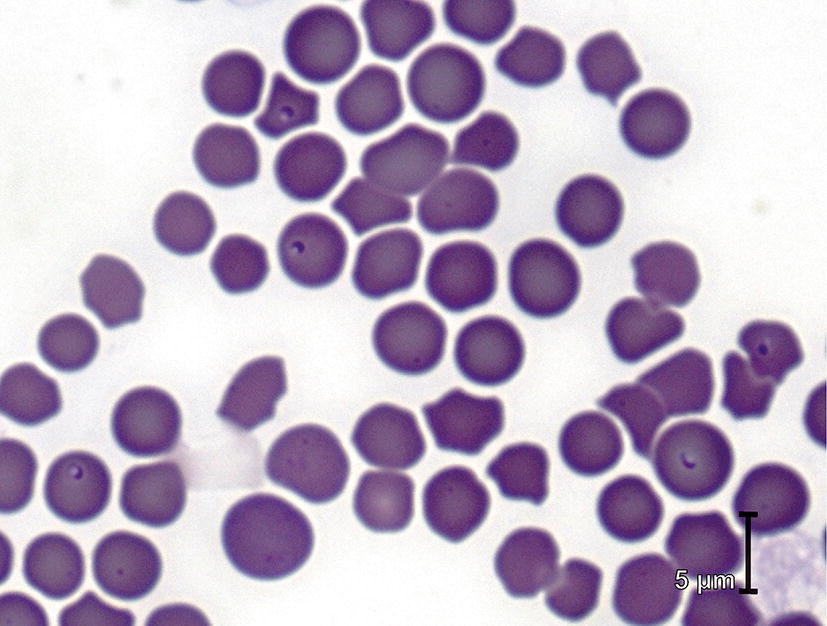
Blood smear from a cat with clinical signs of babesiosis, showing small, intra-erythrocytic *Babesia felis* (*sensu lato*) piroplasms (Courtesy: Dr Sandy Weltan, Vetdiagnostix, Cape Town)

**Fig. 3:**
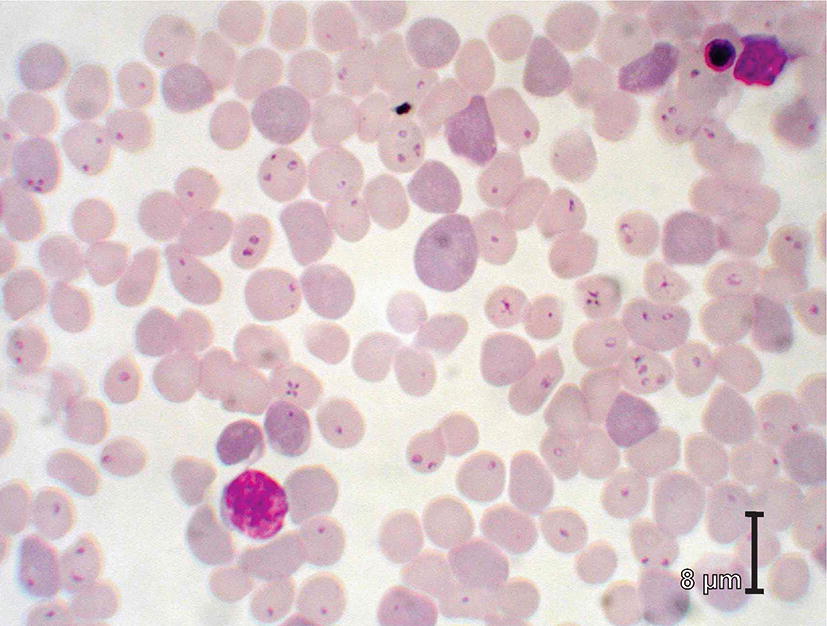
Blood smear from a cat with high parasitaemia of *Babesia felis* (*sensu lato*) piroplasms

Domestic cats can also be infected with other *Babesia* spp. A large, unidentified *Babesia* was incriminated in causing severe clinical signs in a domestic cat in Harare, Zimbabwe [17]. When examining blood smears of sick cats in South Africa, veterinarians occasionally report finding large organisms (Fig. 1), resembling *Babesia rossi* of dogs rather than the small *B. felis* (*s.l.*) (Figs. 2, 3); attempts at identifying these organisms were unsuccessful (pers. obs.). *Babesia canis* subsp. *presentii* was described from two cats in Israel, one a subclinical carrier and the other suffering from co-infection of various other pathogens [18].

*Babesia pantherae*, a large piroplasm isolated from leopards (*Panthera pardus*) in Kenya and *B. herpailuri* isolated from a jaguarundi (*Herpailurus yaguarondi*) originating from Venezuela could be established in domestic cats [19–21]. In both cases overt clinical signs developed only in asplenic cats; spleen-intact cats developed a long-lasting parasitaemia but remained asymptomatic [19]. Unfortunately, this was before the advent of molecular characterisation of piroplasms.

A previous South African survey of cats with clinical signs consistent with babesiosis suggested the presence of further potentially pathogenic piroplasms [15]. Subsequent molecular characterisation revealed that the pathogen involved in two fatal cases of feline babesiosis, one being the first record of cerebral babesiosis in a domestic cat, showed a high similarity with *B. lengau,* previously described from asymptomatic cheetahs [22, 23].

The aim of the present study was to characterise piroplasms from domestic cats in South Africa (Western Cape and KwaZulu-Natal) and Mozambique (Maputo) exhibiting clinical signs of babesiosis, using *18S* rRNA gene sequence data and phylogenetic analysis.

## Methods

**Fig. 4:**
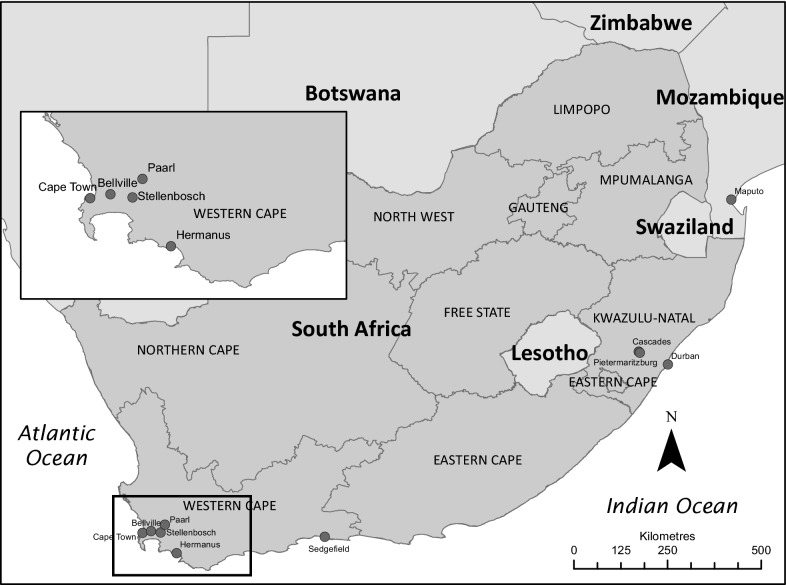
Map of Southern Africa showing the origin of the samples

Blood samples from 18 domestic cats, submitted for diagnostic purposes by private veterinary practitioners to the Department of Veterinary Tropical Diseases, Faculty of Veterinary Science, University of Pretoria, were included in the study (Table 1). Inclusion criteria were clinical signs of babesiosis, identification of piroplasms on blood smears and/or positive reverse line blot (RLB) hybridization assay results. Except for one specimen from Maputo, Mozambique, all samples originated from coastal areas in the Western Cape and KwaZulu-Natal provinces of South Africa (Fig. 4).

**Table 1:** List of domestic cat samples used, with details on the origin, microscopic examination of blood smears, RLB results and phylogenetic classification

Sample ID	Origin	Microscopy	RLB results	No. of clones	Phylogenetic classification
BF221	Cascades, KZN, RSA	*Babesia* spp.	*Babesia* genus-specific only	Not applicable^a^	*B. leo*
BF238	Durban, KZN, RSA	*Babesia* spp.	*Babesia* genus-specific only	Not applicable	*B. leo*
BF272	Hermanus, WC, RSA	No parasites seen	*Babesia* genus-specific only	Not applicable	*B. felis*
BF284	Bellville, WC, RSA	Large *Babesia* spp.	*Babesia* genus-specific only	Not applicable	*B. felis*
BF341	Durban, KZN, RSA	*Babesia* spp.	*Babesia* genus-specific only	Not applicable	*B. leo*
BF342	Bellville, WC, RSA	Large *Babesia* spp.	Negative/Below detection limit	Not applicable	Novel *Babesia* sp. variant 1
BF461	Maputo, Mozambique	*Babesia* spp.	*Babesia* genus-specific only	Not applicable	*B. leo*
BF472	Durban, KZN, RSA	Large *Babesia* spp.	*B. felis*	Not applicable	Novel *Babesia* sp. variant 3
BF475	Durban, KZN, RSA	*Babesia* spp.	*B. felis*	Not applicable	*B. felis*
Cat01	Cape Town, WC, RSA	Large *Babesia* spp.	*B. felis*	2	Novel *Babesia* sp. variant 1
				6	Novel *Babesia* sp. variant 2
Cat02	Cape Town, WC, RSA	*Babesia* spp.	*B. felis*, *B microti*	1	*B. microti*
				5	Novel *Babesia* sp. variant 1
				5	Novel *Babesia* sp. variant 3
Cat03	Cape Town, WC, RSA	*Babesia* spp.	Not tested	5	Novel *Babesia* sp. variant 1
				1	Novel *Babesia* sp. variant 2
Cat05	Sedgefield, WC, RSA	*Babesia* spp.	*B. felis*	8	*B. felis*
Cat06	Sedgefield, WC, RSA	*Babesia* spp.	*B. felis*	9	*B. felis*
Cat07	Pietermaritzburg, KZN, SA	Large *Babesia* spp.	Not tested	1	*B. leo*
Cat08	Paarl, WC, RSA	Large *Babesia* spp.	Not tested	1	Novel *Babesia* sp. variant 2
Cat09	Paarl, WC, RSA	Large *Babesia* spp.	Not tested	1	Novel *Babesia* sp. variant 1
Cat10	Durban, KZN, RSA	*Babesia* spp.	Not tested	1	*B. felis*

^a^PCR amplicon directly sequenced (not subjected to cloning)

*Abbreviations:* KZN, KwaZulu-Natal; RSA, Republic of South Africa; WC, Western Cape Province

DNA was extracted according to the manufacturer’s instructions using the QIAamp^®^ DNA Mini Kit (Qiagen, Whitehead Scientific, South Africa). The V4 hypervariable region of the parasite *18S* rRNA gene was PCR amplified using *Babesia* and *Theileria* genus-specific primers RLB-F2 and biotin-labelled RLB-R2 [24, 25]; PCR reaction conditions were as described by Tembo et al. [26]. DNA extracted from blood from a known *T. parva*-infected buffalo [27] was used as a positive control, while PCR master mix without DNA was used as a negative control. A touch down thermal cycler programme was used to amplify the DNA [25]. The PCR products were then analysed using the RLB hybridization technique as previously described [24, 25, 28, 29]. Genus- and species-specific probes as described by Tembo et al. [26] were included on the membrane; in addition to this, a *B. lengau* probe [22] was also included.

The near full-length parasite *18S* rRNA gene (~1700 bp) was PCR amplified using primers Nbab_1F [30] and TB Rev [31], as previously described by Bosman et al. [22]. Four separate reactions were prepared per sample. Amplicons of all four reactions per sample were pooled to avoid Taq polymerase-induced errors and purified using a QIAquick PCR purification kit (Qiagen, Southern Cross Biotechnology, South Africa) according to the manufacturer’s instructions. Nine of the samples (labelled BF; Table 1) that had been yielded positive RLB results in a previous study [15], were subjected to direct (bi-directional) sequencing on an ABI 3500XL genetic analyser using the amplification primers. For the other nine specimens, PCR amplicons were cloned prior to sequencing (in case of mixed infections not being detected or masked by the RLB assay) into the pGEM-T Easy vector (Promega, Anatech, South Africa) and transformed into competent *Escherichia coli* JM109 cells (JM109 high-efficiency competent cells, Promega). Recombinant plasmids were directly (bi-directional) sequenced on the ABI 3500XL genetic analyser at Inqaba Biotechnical Industries using the vector primers SP6 and T7.

Sequences were assembled and edited using GAP 4 of the Staden package (Version 1.6.0 for Windows) [32]. A search for homologous sequences was performed using BLASTn [33]. The sequences were aligned with sequences of related genera from GenBank using ClustalX (Version 1.81 for Windows). Alignment files were also analysed with CLC Main Workbench version 4.0 (CLC bio, Aarhus, Denmark) to test consistency of the alignment. The alignment was manually truncated to the size of the smallest sequence (1421 bp). The genetic distances between the sequences were estimated by determining the number of nucleotide differences between sequences using MEGA version 7 [34]. Phylogenetic trees were constructed by the Neighbor-Joining (NJ) and Maximum Likelihood (ML) methods as implemented in MEGA 7. The two-parameter model of Kimura [35] was used to construct similarity matrices by single distance from the aligned sequence data; a NJ phylogenetic tree [36] was constructed in combination with the bootstrap method (1000 replicates/tree) [37]. The Hasegawa-Kishino-Yano (HKY + G + I) substitution model [38], determined as the best-fit model using MEGA 7, was used to infer a ML tree in combination with the bootstrap method (1000 replicates/tree) [37]. The *18S* rDNA sequences of *Cardiosporidium ciona* (EU052685), the closest species for which data are available according to Schnittger et al. [39], was included as the outgroup. All consensus trees were edited using MEGA 7. The GenBank accession numbers of reference sequences used in this study are reported in Table 2. The *18S* rRNA gene sequences obtained in this study were submitted to GenBank; the accession numbers are reported in Table 3.

**Table 2:** Accession numbers for GenBank reference sequences used in the present study

GenBank ID	Species	Origin	Host	References
AY072926	*B. canis*	Croatia	Dog	Caccio et al. [52]
AY272047	*B. canis presentii*	Israel	Cat	Baneth et al. [18]
AF158702	*B. conradae*	USA	Dog	Kjemtrup et al. [53]
U16370	*B. divergens*	USA	Cattle	Holman [54]
AF158700	*B. duncani*	USA	Human	Kjemtrup et al. [55]
AF244912	*B. felis*	South Africa	Domestic cat	Penzhorn et al. [40]
AY278443	*B. gibsoni*	Spain	Dog	Criado-Fornelio et al. [56]
GQ411417	*B. lengau*	South Africa	Cheetah	Bosman et al. [22]
KC790443	*B. lengau*	South Africa	Domestic cat	Bosman et al. [23]
KC833036	*B. lengau*	South Africa	Domestic cat	Bosman et al. [23]
AF244911	*B. leo*	South Africa	Lion	Penzhorn et al. [40]
AY452708	*B. leo*	South Africa	Domestic cat	Wuerth (unpubl.)
AB071177	*B. microti* (Munich)	Europe	Human	Tsuji et al. (unpubl.)
AB119446	*B. microti* (Otsu)	Japan	Field rodent	Saito-Ito et al. [57]
AF231348	*B. microti* (GI)	USA	Human	Zahler et al. [58]
AY693840	*B. microti* (Gray)	USA	Human	Slemenda et al. (unpubl.)
XR002459986	*B. microti* (R1)	USA	Human	Cornillot et al. [59]
U16369	*B. odocoilei*	USA	Cervid	Holman et al. [60]
AY661502	*B. odocoilei*	USA	Bighorn sheep	Schoelkopf et al. (unpubl.)
M87565	*B. rodhaini*	Australia	Cell culture	Ellis et al. [61]
DQ111760	*B. rossi*	Sudan	Dog	Oyamada et al. [62]
AY190123	*Babesia* sp. Akita610 Dog tick	Japan	*Ixodes ovatus*	Inokuma et al. [46]
AB251608	*Babesia* sp. MA#230	Japan	Raccoon	Jinnai et al. [44]
KR017880	*Babesia* sp. Maned wolf	USA	Maned wolf	Wasserkrug Naor et al. [42]
AB935172	*Babesia* sp. YA23175	Japan	Raccoon	Komura et al. (unpubl.)
AB935330	*Babesia* sp. SW-R-090616_T1	Japan	Raccoon	Hirata et al. (unpubl.)
AB935331	*Babesia* sp. SW-R-092616_T2	Japan	Raccoon	Hirata et al. (unpubl.)
DQ028958	*Babesia* sp. AJB-2006	USA	Raccoon	Birkenheuer et al. (unpubl.)
KX218429	*Babesia* sp. 1 1093 cl9	Botswana	Lion	McDermid et al. [63]
KX218430	*Babesia* sp. 10 1092 cl9	Botswana	Lion	McDermid et al. [63]
KX218431	*Babesia* sp. 3 1093 cl8	Botswana	Lion	McDermid et al. [63]
KX218432	*Babesia* sp. 4 1093 cl2	Botswana	Lion	McDermid et al. [63]
KX218433	*Babesia* sp. 5 1093 c17	Botswana	Lion	McDermid et al. [63]
KX218434	*Babesia* sp. 6 1092 cl1	Botswana	Lion	McDermid et al. [63]
KX218435	*Babesia* sp. 7 1092 cl3	Botswana	Lion	McDermid et al. [63]
KX218436	*Babesia* sp. 8 1092 cl5	Botswana	Lion	McDermid et al. [63]
KX218437	*Babesia* sp. 9 1093 cl1	Botswana	Lion	McDermid et al. [63]
KX218438	*Babesia* sp. 10 1092 cl9	Botswana	Lion	McDermid et al. [63]
KX218439	*Babesia* sp. 11 1095	Botswana	Lion	McDermid et al. [63]
KX218440	*Babesia* sp. 12 1101	Botswana	Lion	McDermid et al. [63]
AF244913	*Babesia* sp. Strain A Caracal	South Africa	Caracal	Penzhorn et al. [40]
AF244914	*Babesia* sp. Strain B Caracal	South Africa	Caracal	Penzhorn et al. [40]
KF724377	*B*. *venatorum*	China	Human	Sun et al. [64]
AY072925	*B*. *vogeli*	Italy	Dog	Caccio et al. [52]
EU052685	*Cardiosporidium cionae*	–	*Ciona intestinalis*	Ciancio et al. [65]

**Table 3:** Accession numbers for the *18S* rRNA gene sequences generated in the present study

GenBank ID	Sample	Phylogenetic classification	Origin
KC790441	BF461^a^	*B. leo*	Maputo, Mozambique
KC790442	BF472	*Babesia* sp. Variant3	Durban, KZN, RSA
KC790444	BF341A^a^	*B. leo*	Durban, KZN, RSA
KR611115	Cat05_8	*B. felis*	Sedgefield, WC, RSA
KR611116	Cat05_24	*B. felis*	Sedgefield, WC, RSA
KR611117	Cat05_18	*B. felis*	Sedgefield, WC, RSA
KR611118	Cat05_14	*B. felis*	Sedgefield, WC, RSA
KR611119	Cat05_13	*B. felis*	Sedgefield, WC, RSA
KR611120	Cat05_12	*B. felis*	Sedgefield, WC, RSA
KR611121	Cat05_6	*B. felis*	Sedgefield, WC, RSA
KR611122	Cat06_H5	*B. felis*	Sedgefield, WC, RSA
KR611123	Cat06_G5	*B. felis*	Sedgefield, WC, RSA
KR611124	Cat06_D5	*B. felis*	Sedgefield, WC, RSA
KR611125	Cat06_C5	*B. felis*	Sedgefield, WC, RSA
KR611126	Cat06_B6	*B. felis*	Sedgefield, WC, RSA
KR611127	Cat06_A6	*B. felis*	Sedgefield, WC, RSA
KR611128	Cat06_A5	*B. felis*	Sedgefield, WC, RSA
KR611129	Cat06_B5	*B. felis*	Sedgefield, WC, RSA
KR611130	Cat06_F5	*B. felis*	Sedgefield, WC, RSA
KR611131	Cat05_11	*B. felis*	Sedgefield, WC, RSA
KR611132	Cat07_5E	*B. leo*	Pietermaritzburg, KZN, RSA
KR611133	Cat03_5	*Babesia* sp. Variant1	Cape Town, WC, RSA
KR611134	Cat03_10	*Babesia* sp. Variant1	Cape Town, WC, RSA
KR611135	Cat03_3	*Babesia* sp. Variant1	Cape Town, WC, RSA
KR611136	Cat03_1	*Babesia* sp. Variant1	Cape Town, WC, RSA
KR611137	Cat02_3	*Babesia* sp. Variant1	Cape Town, WC, RSA
KR611138	Cat03_9	*Babesia* sp. Variant1	Cape Town, WC, RSA
KR611139	Cat02_6	*Babesia* sp. Variant1	Cape Town, WC, RSA
KR611140	Cat02_10	*Babesia* sp. Variant1	Cape Town, WC, RSA
KR611141	Cat02_12	*Babesia* sp. Variant1	Cape Town, WC, RSA
KR611142	Cat03_8	*Babesia* sp. Variant2	Cape Town, WC, RSA
KR611143	Cat02_4	*Babesia* sp. Variant1	Cape Town, WC, RSA
KR611144	Cat02_2	*Babesia* sp. Variant3	Cape Town, WC, RSA
KR611145	Cat02_1	*Babesia* sp. Variant3	Cape Town, WC, RSA
KR611146	Cat02_9	*Babesia* sp. Variant3	Cape Town, WC, RSA
KR611148	Cat02_13	*Babesia* sp. Variant3	Cape Town, WC, RSA
KR611149	Cat01_G	*Babesia* sp. Variant1	Cape Town, WC, RSA
KR611150	Cat01_K	*Babesia* sp. Variant2	Cape Town, WC, RSA
KR611151	Cat01_F	*Babesia* sp. Variant1	Cape Town, WC, RSA
KR611152	Cat01_A	*Babesia* sp. Variant2	Cape Town, WC, RSA
KR611153	Cat01_J	*Babesia* sp. Variant2	Cape Town, WC, RSA
KR611154	Cat01_I	*Babesia* sp. Variant2	Cape Town, WC, RSA
KR611155	Cat01_B	*Babesia* sp. Variant2	Cape Town, WC, RSA
KR611156	Cat01_E	*Babesia* sp. Variant2	Cape Town, WC, RSA
KR611158	Cat08_13	*Babesia* sp. Variant2	Paarl, WC, RSA
KR611159	Cat02_4b	*Babesia* sp. Variant3	Cape Town, WC, RSA
KR732967	BF475	*B. felis*	Durban, KZN, RSA
KR732968	BF284	*B. felis*	Bellville, WC, RSA
KR732969	BF272	*B. felis*	Hermanus, WC, RSA
KR732970	BF342	*Babesia* sp. Variant1	Bellville, WC, RSA
KR732971	BF461A^a^	*B. leo*	Maputo, Mozambique
KR732972	BF341^a^	*B. leo*	Durban, KZN, RSA
KR732973	BF238	*B. leo*	Durban, KZN, RSA
KR732974	BF221	*B. leo*	Cascades, KZN, RSA
KT182985	Cat10_14_11	*B. felis*	Durban, KZN, RSA
KT182986	Cat9_8	*Babesia* sp. Variant1	Paarl, WC, RSA
MK095342	46Cat02_10b^b^	*B. microti*	Cape Town, WC, RSA
MK095343	46Cat02_10b_new^b^	*B. microt*	Cape Town, WC, RSA

^a^Duplicate samples received per animal (BF341 and 461)

^b^Sequences derived from the same clone (46Cat02_10b and 46Cat02_10b_new)

*Abbreviations*: KZN, KwaZulu-Natal; RSA, Republic of South Africa; WC, Western Cape Province

## Results

Clinical reports indicated that 15 cats showed severe clinical signs of babesiosis, e.g. lethargy, anaemia, icterus and fever. Although no detailed clinical reports were available for three cats (BF341, BF472 and BF455) (Table 1), the attending veterinarians had made tentative diagnoses of babesiosis. With the exception of one cat (BF272), organisms morphologically consistent with piroplasms were seen on microscopic examination of blood smears from 17 of the cats; seven of these had been reported as a “large” *Babesia* (Table 1).

The RLB hybridization assay results revealed that of the 13 samples tested, six (46.2%) tested positive for the presence of *B. felis* DNA. One of these samples (Cat02) had a mixed species infection with *B. microti* (Table 1). This was subsequently confirmed by cloning and sequencing analysis of the *18S* rRNA gene. PCR amplicons from a further six samples (46.2%) hybridized with the *Babesia* genus-specific probe only, suggesting the presence of a potentially novel *Babesia* species. One sample (BF342) tested negative or below the detection limit of the assay although a large *Babesia* had been observed by microscopy.

A total of 55 nearly full-length (1484–1525 bp) parasite *18S* rRNA gene sequences were obtained from the 18 samples. Of these, nine were directly sequenced and the rest were cloned prior to sequencing, yielding a further 46 sequences from the clones (Table 1). A BLASTn search revealed that sequences from six cats (two of from Durban, KwaZulu-Natal, and four from the Western Cape) were most closely related to a published *18S* rRNA gene sequence of *B. felis* (AF244912) which was previously described from a domestic cat and caused severe clinical babesiosis in naturally and experimentally infected cats in South Africa [11, 13]. One of these 21 sequences (Cat06_A6) had 100% sequence identity to the published *B. felis* sequence, while the remaining sequences had 99% identity, differing by one nucleotide from the published *B. felis 18S* rRNA gene sequence over a 1525 bp region.

Sequences from four cats had 100% identity with published *B. leo* sequences, while one sequence (Cat07_5E) had 99% identity with *B. leo* (with a 3 nucleotide difference over a 1520 bp region). *Babesia leo* was previously described from lions in the Kruger National Park, South Africa, and was shown to be a distinct species from *B. felis* and other felid piroplasms [40]. One specimen was from Maputo, Mozambique, the other four being from KwaZulu-Natal, i.e. all on the north-eastern seaboard of southern Africa.

One sequence (Cat02new) had 98–100% sequence identity with published *B. microti 18S* rRNA gene sequences, including strains from the zoonotic *B. microti* lineages (USA, Munich, Kobe and Otsu/Hobetsu from Japan). It had 100% sequence identity to the published *B. microti* Otsu strain (AB119446) and differed by 3–6 nucleotides from the *B. microti* Gray (AY693840) and *B. microti* Munich (AB071177) strains, respectively.

The most interesting finding, however, was that sequences obtained from seven cats, six from the Western Cape Province and one from Durban, KwaZulu-Natal, constituted a novel *Babesia* group with 96% identity to *Babesia* spp. previously described from captive maned wolves (*Chrysocyon brachyurus*) [41, 42], raccoons (*Procyon lotor*) from the USA [43] and Japan [44, 45] and from ticks collected from dogs in Japan [46]. Three genetic variants were identified within this novel *Babesia* group (designated “Novel *Babesia* sp. genetic variants 1, 2 and 3”), differing by 1 to 3 nucleotides from each other. Genetic variant 1 was found in five cats, variant 2 in three cats and variant 3 in two cats (Table 2). Three cats were infected with two genetic variants: two with variants 1 and 2, and one with variants 1 and 3.

**Fig. 5:**
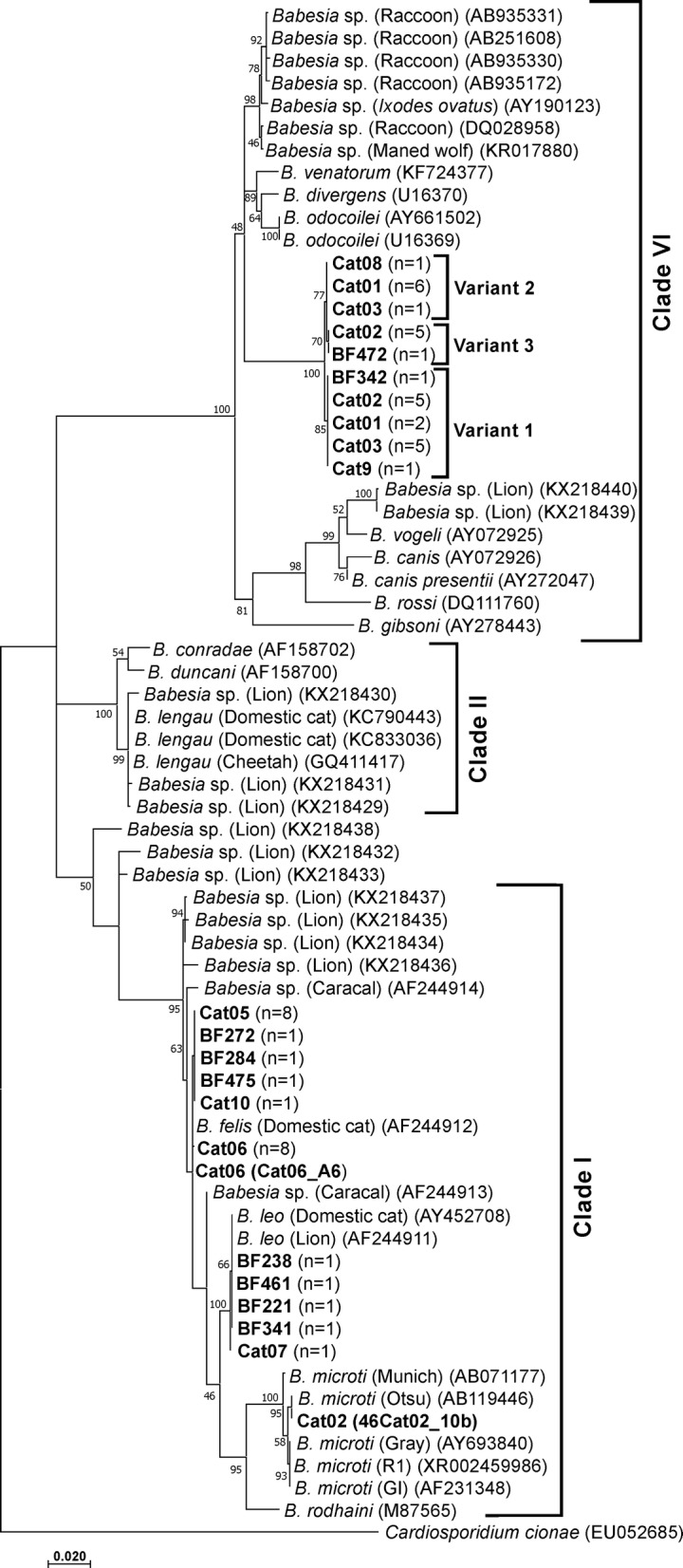
Maximum likelihood tree showing the evolutionary relationships of the *Babesia 18S* rDNA sequences obtained, with published sequences. The evolutionary history was inferred by using the Maximum Likelihood method based on the Hasegawa-Kishino-Yano model [38]. A discrete Gamma distribution was used to model evolutionary rate differences among sites [5 categories (+G, parameter = 0.4367)]. The rate variation model allowed for some sites to be evolutionarily invariable [(+I), 57.75% sites]. The tree is drawn to scale, with branch lengths measured in the number of substitutions per site. All positions containing gaps and missing data were eliminated. There were a total of 1208 positions in the final dataset. Evolutionary analyses were conducted in MEGA7 [34]

The observed sequence similarities were subsequently confirmed by phylogenetic analyses. NJ and ML analyses were used to reveal the phylogenetic relationships between the near full-length *18S* rRNA gene sequences obtained from this study to related *Babesia* species previously deposited in GenBank (Table 1). The topologies of both trees were similar. The ML tree is shown in Fig. 5. Three distinct clades, in concordance with Schnittger et al. [39], were obtained representing Clade I (including rodent-infecting *B. microti* and *B. rodhaini*, and feline-infecting *B. leo* and *B. felis* parasites), Clade II (including *B. duncani* isolated from humans, canine *B. conradae* and *B. lengau* described from cheetah in South Africa) and Clade VI (*Babesia* (*s.s.*), including the canine-infecting *B. gibsoni*, *B. canis*, *B. rossi* and *B. vogeli*, the human-infecting isolate *B. venatorum*, as well as species infecting ungulates (such as *B. divergens* and *B. odocoilei*) and recently described *Babesia* species infecting other carnivores such as bears, cougars and raccoons, as well as field rodents. The novel *Babesia* species identified in this study grouped within Clade VI, also referred to as the “carnivore/rodent clade” by Schnittger et al. [39].

## Discussion

The *B. felis*-positive specimens were from both the Western Cape (*n* = 4) and KwaZulu-Natal (*n* = 2). There is a single report of *B. leo* from a sick cat, but it was a mixed infection with *B. felis* [15]. The results of the present study unambiguously implicate *B. leo* in causing clinical babesiosis in domestic cats. The *B. leo*-positive specimens were all from the north-eastern seaboard of southern Africa: KwaZulu-Natal (*n* = 4) and Maputo, Mozambique (*n* = 1), which constituted the first record of *B. leo* from that country. The Kruger National Park, South Africa, from where *B. leo* was first described [40], has a 320-km-long border with Mozambique. In a direct line, the south-eastern tip of the Park is only *c*.70 km from Maputo.

Sequence and phylogenetic analysis of the *18S* rRNA gene from seven cats showed that they harboured a novel *Babesia* sp. which segregated into three separate genetic variants in *Babesia* clade VI, the carnivore/rodent clade [39]. *Babesia felis*, *B. leo* and *B. lengau*, the three South African felid piroplasms hitherto known to cause clinical signs in domestic cats, were relatively closely related [15, 22, 40]. In contrast, the novel *Babesia* sp. reported here had only 92% sequence identity with *B. felis* (AF244912) and 89% sequence identity with *B. leo* (AF244911) and *B. lengau* (KC790443 and GQ411417), respectively. The *18S* rRNA gene has been widely used to characterize and classify previously unknown *Theileria* and *Babesia* parasites [24, 25, 30, 47–49]. It has, however, not been established to what extent *18S* rRNA gene sequences must differ for the source organisms to be considered different species, rather than merely a genetic variant or genotype within a species [50, 51]. A single gene tree does not necessarily reflect a species tree [39]; therefore, a tree should ideally be constructed using multiple genotypic characters of potentially different evolutionary histories [39].

The novel genetic variants reported here were most closely related (96% identity) to a novel *Babesia* sp. reported from culled feral raccoons from Japan [44, 45] and from a clinically affected juvenile raccoon from the USA [43]. It is tempting to speculate that feral raccoons may also have been the source of an incidental finding of this *Babesia* sp. in ticks collected from healthy dogs in Japan [46]. The same *Babesia* sp. was incriminated in causing severe clinical babesiosis in two South American maned wolves from the same zoological park in Kansas, USA [41, 42].

When examining blood smears, veterinarians described the novel genetic variants reported here as “large” babesias. This may be the elusive large *Babesia* reported from cats in southern Africa. The arbitrary classification of babesias as either “large” or “small” is not satisfactory, however. For instance, the abovementioned *Babesia* sp. from raccoons was reported to be closely related to *B. odocoilei* and *B. divergens* [44], both generally regarded as “large” species. Nevertheless, the mean length of the round, oval, amoeboid or piriform organisms was 3.13 ± 0.77 µm (range 1.25–4.8 µm) and the mean width was 2.5 ± 0.61 µm [45]. Round, oval and amoeboid forms are trophozoites, which can be expected to increase in size. For comparative purposes, measuring newly formed merozoites should give more consistent and reliable results.

Six of the seven specimens of the novel genetic variants were from a fairly restricted area in the Western Cape Province (Bellville, Cape Town and Paarl). The other case was from Durban, KwaZulu-Natal. No further information was known about the latter case, e.g. whether the cat may originally have come the Cape Town area. It may be possible that the natural hosts and/or vectors of these novel genetic variants are restricted to the Western Cape Province. Due to lacking an appropriate type specimen, we refrain from describing a new species but refer to the novel organism as *Babesia* sp. cat Western Cape.

Further characterisation of this novel organism is warranted to understand the pathogenesis and epidemiology, as well as to develop appropriate diagnostic markers. Obtaining appropriate specimens poses a challenge, however. Veterinarians in the feline babesiosis-endemic area usually confirm a diagnosis by finding piroplasms on a blood smear and then treat the cat. Blood specimens are only rarely submitted for confirmation of a diagnosis. Furthermore, our laboratory is in Pretoria, *c*.600 km from Durban and 1500 km from Cape Town, which hampers routine sampling of clinical cases.

## Conclusions

Our results indicate the presence of a novel potentially pathogenic *Babesia* sp. in felids in South Africa, which is not closely related to *Babesia felis*, *Babesia lengau* and *Babesia leo*, the three species known to be pathogenic to cats. Due to the lack of an appropriate type-specimen, we refrain from describing and a new species but refer to the novel organism as *Babesia* sp. Cat Western Cape.
